# Supplementary material for: An improved demand curve for analysis of food or drug consumption in behavioral experiments
Source: Psychopharmacology (Berl). 2020 Mar 13;237(4):943–55. doi: 10.1007/s00213-020-05491-2 (PMC7113227; doi:10.1007/s00213-020-05491-2)
Supplement: Supplementary file 1 — (PDF 195 kb) [file 213_2020_5491_MOESM1_ESM.pdf]

Noname manuscript No.
(will be inserted by the editor)

An improved demand curve for analysis of food or drug consumption in behavioral experiments: Supplementary information

Mark Newman · Carrie R. Ferrario

1 Software for demand curve analysis

In the accompanying online materials (`http://umich.edu/~mejn/demandcurve`) we provide a software program that performs the analyses described in the main paper using the proposed demand curve of Eq. (20). Here we describe the use of this program. The same information can also be found in the form of a video tutorial, also in the accompanying materials.

1.1 Installation

The program is called `demand.py`. To install it, simply download it from the web address given above and place it in the folder or directory containing the data you wish to analyze.

The program is written in the Python computer language. Running it requires that you also have the Python language installed. Many computers come with Python already installed, but if yours does not, Python is free to download from the web. There are various versions available, but we recommend the "Anaconda" Python distribution, which contains everything you will need in one single package. Anaconda is available for free from `www.anaconda.com`. Two versions are currently available, version 2 (actually version 2.7 at the time of writing) and 3 (version 3.7 at the time of writing). Our program will work with either but we recommend installing version 3 simply because it is the most up-to-date.

If you wish to install Python by another route (for instance using a package manager), then you should, at a minimum, install the Python language itself (either version 2 or version 3), plus the packages "scipy" (for curve fitting) and "matplotlib" (for graphics).

Mark Newman
Center for the Study of Complex Systems, University of Michigan, Ann Arbor, Michigan, USA

*Carrie R. Ferrario (corresponding author, `ferrario@umich.edu`)
Department of Pharmacology, University of Michigan, Ann Arbor, Michigan, USA

	A	B	C	D
1	1.39	5	5	6
2	2.5	10	8	8
3	4.35	12	13	16
4	7.69	19	20	22
5	13.89	28	35	39
6	25	31	37	40
7	43.48	37	31	19
8	76.92	22	15	10
9	142.86	8	8	4
10	250	4	0	2
11				
12				

Fig. S1 Example of the format of the input data. The first column (column A) represents prices, which can be in any units. (Ours are in responses per milligram.) Second and subsequent columns, of which there can be any number, represent responses at each price point for separate animals, sessions, or data sets. In this case the file has columns for three different sessions (columns B, C, and D). Our responses are whole numbers, since they represent lever presses by individual animals, but one can also use responses averaged over several animals or sessions, in which case the values need not be whole numbers. When complete, the spreadsheet should be saved in CSV (comma-separated value) format with commas as separators between the fields.

1.2 Data input

The program takes input data in the form of prices and responses. It can handle an arbitrary number of price points in a single analysis and can analyze data from multiple animals or multiple sessions in a single run. Data should be prepared in a spreadsheet as shown in Fig. S1. The first column of the sheet contains the prices, in any units you choose. Prices can be in any order—they need not be in increasing (or decreasing) order, though they can be. There must be at least four different prices for the program to work correctly (i.e., at least four rows in the spreadsheet), since there are four parameters in the fitted demand curve. (The number of data points for any fit can never be less than the number of parameters.) The second and subsequent columns contain the response at each price in terms of the amount of work (e.g., number of lever presses) done in experimental sessions, with one column for each session or data set. There can be just a single session (for a total of two columns—prices and responses) or many (for a total of $n + 1$ columns if there are n sessions). There should be no blank cells, nor empty rows or columns, in the spreadsheet, except for the unused rows and columns below and to the right of the data. (If you wish to analyze data from experiments using different numbers of price points then you will have to use separate spreadsheets.)

The spreadsheet should be saved in CSV (comma-separated value) format, using commas as separators between the values and the file extension "`.csv`". (Note that CSV files can be saved with spaces as separators, but this should be avoided as it will not work with our program.) When using Microsoft Excel, for instance, saving as a CSV file is a standard option under the "Save as" menu item; any of the sub-formats listed there (Macintosh, MS-DOS, or CSV) will work. An example input file, called `example.csv`, is included in the accompanying online materials.

1.3 Running the program

Windows: On an appropriately configured Windows system it will be possible to run the program from a command prompt window (also known as a "DOS window"), by changing to the folder where the program file resides and typing either "`py demand.py`" or "`python demand.py`" into the command window (depending on how the computer is set up). Alternatively, the program can be run inside a Python development environment such as Jupyter, Idle, or Spyder. The latter are all included, for instance, in the Anaconda distribution mentioned above. When Anaconda is started it will display a screen offering a choice of environments for use. Start the environment of choice by double-clicking on its launch icon, then load the program `demand.py` and run it. Specific procedure will depend on which environment you use. See the video tutorial for an example using the Spyder environment.

OSX: On a Mac one can normally open a command window and type "`python demand.py`" to run the program, or use any of the several available Python development environments—see the instructions for Windows users above.

Linux: Under Linux one can run the program from the command line with the command "`python demand.py`" or from within a development environment.

1.4 Using the program

When first run, the program will ask for the name of the input data file. Type in the name of the CSV file containing your data. (The name is case-sensitive, so take care with capitalization.) The file extension "`.csv`" at the end of the name is optional and can be omitted. The file name can also be provided to the program as a command-line argument when the program is run. For instance, under Windows one might type "`py demand.py example.csv`" and the program would use the data file `example.csv`. This is a convenient feature when running the program in batch mode (i.e., unattended).

Once the file name is entered, the program will perform the analysis. It works by computing consumption levels from the response data then performing a nonlinear least-squares fit of the logarithms of the consumption values to Eq. (21) from the main paper. The program automatically calculates parameter values and a range of other quantities, and takes appropriate precautions so that, for instance, parameter values cannot become infinite.

The output of a typical run of the program looks like this:

```
Data read from file example.csv
  Price points: 10
  Curves:       3

Analyzing curve 1
Analyzing curve 2
Analyzing curve 3
```

	A	B	C	D	E	F	G	H
1	CURVE	Q0	P0	a	b	Rmax	Pmax	Normalized Pmax
2	1	3.73479880041	66.7556021893	3.62425289705	1.06517621866	33.5980887878	26.9848642436	100.783038606
3	2	3.23071824185	25.8486530742	2.37193733677	2.01969792201	37.540776139	22.1024534254	71.4067994711
4	3	3.51759956781	18.9630901245	2.37188552895	3.41691227453	41.5827489978	17.2870703917	60.8089913388
5								

Fig. S2 Example of the output spreadsheet generated by the program. Columns contain the parameters Q_0, P_0, a, and b of the fitted demand curves, plus the derived values $R_{\max}$, $P_{\max}$, and $\widetilde{P}_{\max}$ ("Normalized $P_{\max}$"), as indicated, and there is one row of the spreadsheet for each data set in the input file.

The program gives a summary of the data it found in the input file (number of price points and number of data sets), then lists the demand curves one by one as it analyzes them. The program may also print out cautionary messages if it believes there are potential problems with the data. For instance, if the fitted value of Q_0 lies well outside the range of observed consumption it will print a message like this:

```
Caution: Q0 = 880 is substantially outside the data range [2.5 to 140]
```

This could be an indication that the experiment failed to probe the salient portion of the demand curve near the turning point at which demand falls off.

When the program finishes, which typically takes a few seconds, it will produce a set of output files in the same folder. First, there will be an output data file. If the input file was called `example.csv`, the output file will be called `example_params.csv`. This is a CSV spreadsheet containing the best fit values of the parameters Q_0, P_0, a, and b for each session in the input file, plus the values of the derived quantities $R_{\max}$, $P_{\max}$, and $\widetilde{P}_{\max}$. This file can be opened in Microsoft Excel, Google Docs, or any similar spreadsheet program. There is one row of the file for each session, in the same order as the columns of the input file, so that the row for curve 1 corresponds to data in column B of the input file, curve 2 to data in column C, and so forth. An example output file is shown in Fig. S2.

Second, the program produces a set of output figure files, one for each input session or data set (i.e., for each column from B onward in the original input file). An example is shown in Fig. S3. Each figure file contains two graphs: the left graph shows, as a function of price, the data points for consumption in blue and the fitted demand curve in green; the right graph shows the original data for responses in blue and the fitted curve in green. If the input data file is called `example.csv` then these figure files will have names `example_1.png`, `example_2.png`, and so forth, numbered in the same order as the columns of the input file.

It is a good idea before using the numerical results of any of the fits to inspect the corresponding figures to make sure that the fit is a reasonable one. If the data fluctuate a lot, or if the experiment fails to probe the correct price range, then the fit may be a poor one. In cases where the data are entirely unlike the expected demand-curve form of plateau-plus-decline, the fit may fail and the fitted curve will not resemble the data points. Some thought may be needed to interpret the results of the procedure in any specific case.

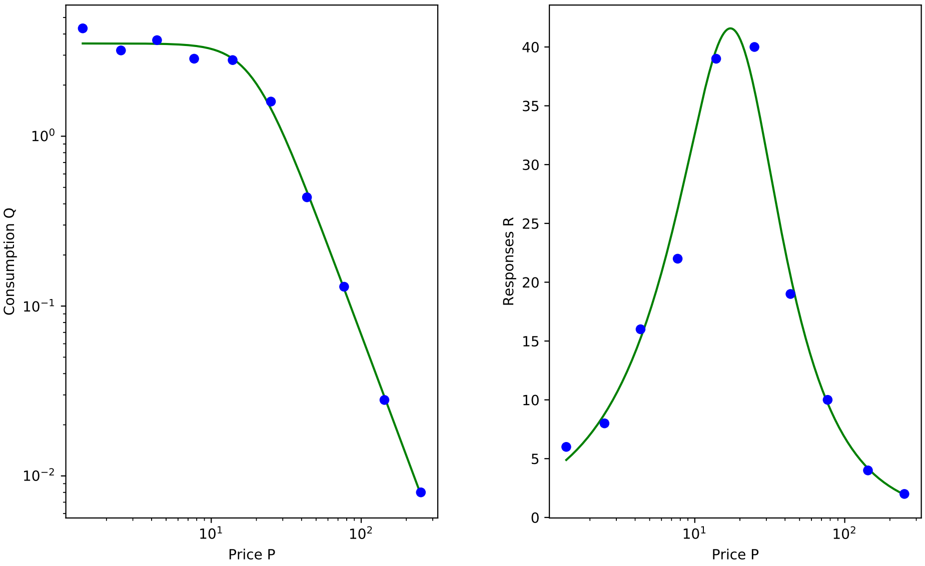

Fig. S3 A typical figure produced by the program. Left: data for consumption against price (points) and the best fit of the demand curve from Eq. (20) of the main paper, as determined by the program (solid line). Right: the original response data against price (points) and the corresponding revenue curve determined from the demand curve using Eq. (4) (solid line). R_{max} is the highest value of the revenue curve and P_{max} is the price at which that highest value occurs. In this case we find $R_{\text{max}} = 41.6$ responses and $P_{\text{max}} = 17.3$ responses per milligram.

1.5 Initial parameter values

In performing a fit, the program works by guessing approximate initial values of the four parameters Q_0, P_0, a, and b and then adjusting them repeatedly until a good fit is achieved. While the program normally makes sensible initial guesses for the parameters, there may be occasional instances, particularly with poor-quality data, where the process fails because the initial guesses are too far from the correct values, and the program will not generate a sensible fit. For advanced users, we incorporate an additional feature in the program that allows the user to choose the starting values themselves, overriding the values chosen by the program. To use this feature the program should be started from a command line or DOS window and the desired starting values of Q_0, P_0, a, and b should be given, in that order, on the command line, after the name of the CSV file. Thus, for instance, on a suitably configured Windows system one might type the line "`py demand.py example.csv 3.5 30 3 2`". This tells the program to read data from the file `example.csv` and initially to set the parameters (for all sessions) to the values $Q_0 = 3.5$, $P_0 = 30$, $a = 3$, and $b = 2$. In some cases this will allow the program to find a fit to the data that it would otherwise not be able to find.

How should one go about choosing suitable starting values for the parameters when using this feature? In the case of Q_0, a plot of consumption against price, as in the left panel of Fig. S3, is often enough to make a useful estimate—one simply looks for the height of the plateau on the left-hand side of the figure. In

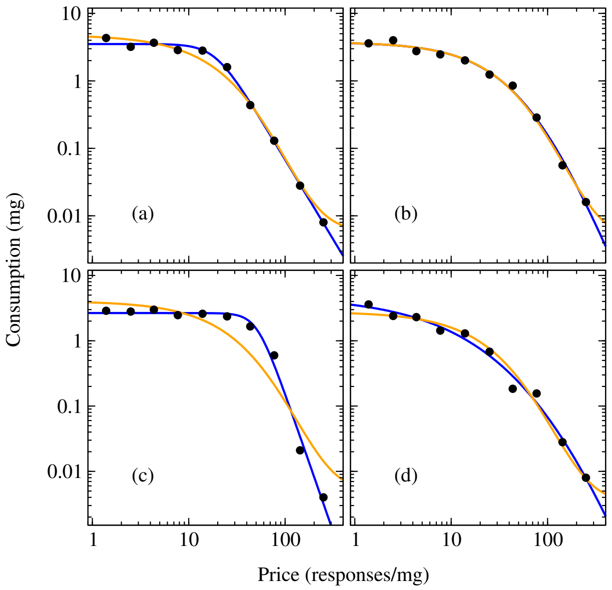

Fig. S4 The same cocaine self-administration data shown in Fig. 5 of the main paper, along with best-fit curves using the form proposed in this paper, Eq. (21) (blue curves), and the exponential form of Eq. (9) (orange curves).

Fig. S3, for example, a value of about $Q_0 = 3$ appears sensible. Similarly for P_0 one looks for the price at which the consumption starts to drop off—a value of about $P_0 = 20$ looks reasonable for Fig S3. The parameters a and b can be harder to estimate, but one can often find good values by trial and error. Typical values for a are between about 1 and 5, and typical values for b are between about 0.5 and 20. In the most difficult cases it may be necessary to run the program more than once with the same data and different starting parameters to find values that work well.

2 Additional analysis

Figures 1 and 2 in the paper show fits of the same cocaine self-administration data to the demand curve form we propose and to the exponential form of Hursh and Silberberg (2008). In Fig. S4 we show a further comparison of fits to these two forms. The figure is a modified version of Fig. 5 from the paper, showing the same data for cocaine self-administration by four different male rats and the same fits to the form we propose (blue curves). But now we also add best fits to the exponential form, with the parameter k once again set equal to the maximum range of the logarithm of the data over all sessions, which in this case results in $k = 6.62$. As the figure shows, the fit to the exponential form is about as good as to our proposed form in two cases (b and d), a little worse in one case (a), and significantly poorer in the last case (c).

Figure 5 in the paper shows fits of our proposed demand curve form to data for cocaine self-administration by four male rats. The data shown are relatively

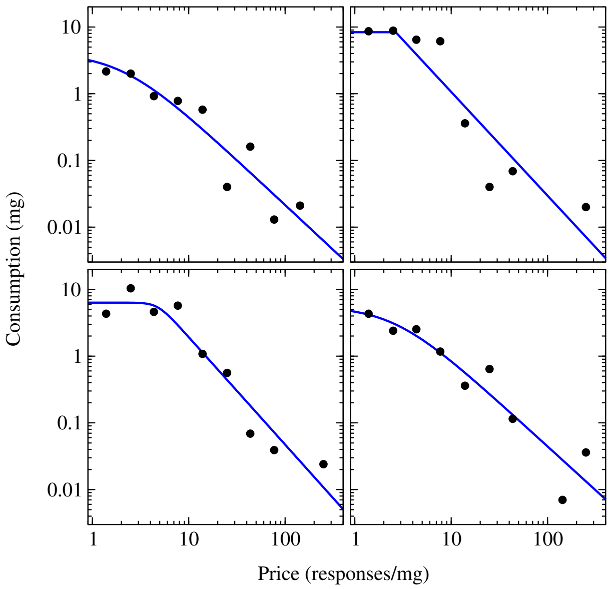

Fig. S5 Cocaine self-administration data for four more male rats, taken from the same experiment as Fig. S4, but this time deliberately picking data of poorer quality, in order to test our approach. As the figure shows, our proposed demand curve form, as implemented in the software described here, is still able to find convincing fits to the data. (Previously unpublished data kindly provided by C. Carr and T. E. Robinson.)

clean examples in which the demand roughly follows the expected plateau-plus-decline form. In every experiment, however, there are always some sessions in which animals do not behave as expected and the data are less clean—sometimes much less so. In Fig. S5 we show four more examples from the same set of cocaine self-administration experiments, but in this case we have deliberately chosen data of poorer quality, showing the largest deviation from the expected behavior. As the figure shows, our procedure (implemented in the software described above) is still able to find convincing fits to the data, allowing us to extract values for parameters such as $P_{\max}$ or $R_{\max}$.
